# Adaptive Regulation of Osteopontin Production by Dendritic Cells Through the Bidirectional Interaction With Mesenchymal Stromal Cells

**DOI:** 10.3389/fimmu.2018.01207

**Published:** 2018-06-01

**Authors:** Sara Scutera, Valentina Salvi, Luisa Lorenzi, Giorgia Piersigilli, Silvia Lonardi, Daniela Alotto, Stefania Casarin, Carlotta Castagnoli, Erica Dander, Giovanna D’Amico, Silvano Sozzani, Tiziana Musso

**Affiliations:** ^1^Department of Public Health and Pediatric Sciences, University of Turin, Turin, Italy; ^2^Department of Molecular and Translational Medicine, University of Brescia, Brescia, Italy; ^3^Skin Bank, Department of General and Specialized Surgery, A.O.U. Citta della Salute e della Scienza di Torino, Turin, Italy; ^4^“M. Tettamanti” Research Center, Pediatric Department, University of Milano-Bicocca, Monza, Italy

**Keywords:** dendritic cells, mesenchymal stromal cells, osteopontin, ccl5, adipogenesis, osteogenesis

## Abstract

Mesenchymal stromal cells (MSCs) exert immunosuppressive effects on immune cells including dendritic cells (DCs). However, many details of the bidirectional interaction of MSCs with DCs are still unsolved and information on key molecules by which DCs can modulate MSC functions is limited. Here, we report that osteopontin (OPN), a cytokine involved in homeostatic and pathophysiologic responses, is constitutively expressed by DCs and regulated in the DC/MSC cocultures depending on the activation state of MSCs. Resting MSCs promoted OPN production, whereas the production of OPN was suppressed when MSCs were activated by proinflammatory cytokines (i.e., TNF-α, IL-6, and IL-1β). OPN induction required cell-to-cell contact, mediated at least in part, by β1 integrin (CD29). Conversely, activated MSCs inhibited the release of OPN *via* the production of soluble factors with a major role played by Prostaglandin E_2_ (PGE_2_). Accordingly, pretreatment with indomethacin significantly abrogated the MSC-mediated suppression of OPN while the direct addition of exogenous PGE_2_ inhibited OPN production by DCs. Furthermore, DC-conditioned medium promoted osteogenic differentiation of MSCs with a concomitant inhibition of adipogenesis. These effects were paralleled by the repression of the adipogenic markers PPARγ, adiponectin, and FABP4, and induction of the osteogenic markers alkaline phosphatase, RUNX2, and of the bone-anabolic chemokine CCL5. Notably, blocking OPN activity with RGD peptides or with an antibody against CD29, one of the OPN receptors, prevented the effects of DC-conditioned medium on MSC differentiation and CCL5 induction. Because MSCs have a key role in maintenance of bone marrow (BM) hematopoietic stem cell niche through reciprocal regulation with immune cells, we investigated the possible MSC/DC interaction in human BM by immunohistochemistry. Although DCs (CD1c^+^) are a small percentage of BM cells, we demonstrated colocalization of CD271^+^ MSCs with CD1c^+^ DCs in normal and myelodysplastic BM. OPN reactivity was observed in occasional CD1c^+^ cells in the proximity of CD271^+^ MSCs. Altogether, these results candidate OPN as a signal modulated by MSCs according to their activation status and involved in DC regulation of MSC differentiation.

## Introduction

Mesenchymal stromal cells (MSCs) are non-hematopoietic precursors able to self-renew, which differentiate into multiple cell lineages including osteoblasts, chondrocytes, and adipocytes (1). Originally isolated from the bone marrow (BM), MSCs are present in multiple tissues including adipose tissue and umbilical cord blood (2). Beside their regenerative capacity, MSCs possess immunomodulatory properties (3, 4) raising their importance as a potential therapeutic strategy in immune-related diseases (5, 6). Indeed, MSCs suppress the proliferation of T cells and the differentiation/maturation of antigen-presenting cells. They also induce regulatory T cells that further suppress immune responses (7–9). MSCs were reported to inhibit the effector functions of other immune cells, including B and NK cells by a cell-to-cell contact mechanism and the secretion of soluble factors (10, 11).

The immunomodulatory action of MSCs apparently depends on the local microenvironment. At the sites of inflammation, IFN-γ and TNF-α are key cytokines in licensing MSCs to become immunosuppressive. On the other hand, in the absence of inflammation, MSCs can stimulate immune responses (4). For example, despite their ability to inhibit the proliferation of activated T cells, MSCs can support T cells as well as neutrophils survival in the BM (12–14). While MSCs can modify immune cell behavior, a reciprocal influence of immune cells on MSC functions was also reported with a role of immune cells in MSC homeostasis and in the process of tissue regeneration (15).

Dendritic cells are professional antigen-presenting cells that play a critical role in the induction of both immunity and tolerance (16–18). Given the pivotal role of DCs in immunity, the influence of MSCs on DC functions was investigated in several studies (19). MSCs were reported to significantly impair DC differentiation and maturation and to inhibit the secretion of several cytokines, such as TNF-α and IL-12 (20–22). Coherently with an overall anti-inflammatory effect, MSCs colocalize with DCs at the sites of inflammation (23). However, MSCs also colocalize with DCs in the perivascular areas of healthy adipose tissue where DCs concur to tissue homeostasis (24). In addition, MSCs and DCs colocalize in the BM in perisinusoidal areas (25, 26). Thus, the interplay between DCs and MSCs might also happen in homeostatic conditions.

We and others have previously shown that DCs are a prominent source of osteopontin (OPN) (27, 28), a multifunctional protein that influences both immune and non immune cells. OPN functions through the interaction with multiple cell surface receptors known to be expressed by MSCs, such as various integrins and CD44 (29, 30). Under physiological conditions, OPN expression is restricted to certain tissues including bone, kidney, and intestine where it accomplishes a physiologic control of bone remodeling and hematopoietic stem cell location and proliferation (31, 32). Conversely, in inflamed and injured tissues, OPN is strongly upregulated and is involved in the pathogenesis of various inflammatory disorders, such as autoimmune disorders, several types of cancer, and cardiovascular diseases (29, 33, 34). Indeed, OPN was shown to regulate innate and adaptive immune responses and is generally classified as a proinflammatory cytokine even though it also has antinflammatory actions (35, 36). In addition, OPN regulates MSC migration and differentiation to promote wound healing (37). These OPN properties prompted us to investigate the role of OPN in the interplay between MSCs and DCs in both homeostatic and inflammatory conditions. The results here reported show that OPN is modulated in the DC/MSC crosstalk and plays a role in the MSC differentiation mediated by DCs.

## Materials and Methods

### Generation of Human MSC From Adipose Tissue

Human adipose tissues were collected by lipoaspiration from healthy donors after written consent and according with the Declaration of Helsinki and with the local ethic committee (Comitato Etico Interaziendale A.O.U. Città della Salute e della Scienza di Torino—A.O. Ordine Mauriziano—ASL TO1, number 0009806). MSCs were obtained after a monolayer expansion of the stromal vascular fraction (SVF) isolated from adipose tissue samples as previously described (38, 39). Briefly, the SVF cells were seeded in T25 flasks and cultured in DMEM with 10% FBS, 2 mM glutamine, and 1% antibiotics (Gibco, Thermo Fisher Scientific, Waltham, MA, USA) and the medium was replaced to eliminate non-adherent cells after 24 h. Then MSCs were cultured for 2–3 passages, and their phenotype was analyzed by flow cytometry. MSCs were identified as CD73, CD90, and CD105 positive cells and negative for the CD11b, CD34, and CD45 expression.

### Monocyte-Derived DC Preparation

Monocytes were isolated from peripheral blood mononuclear cells obtained from healthy donor buffy coats (through the courtesy of the S.C. Centro Produzione e Validazione Emocomponenti, Torino) by immunomagnetic selection with CD14 microbeads (MACS monocyte isolation kit from Miltenyi Biotec, Bergisch Gladbach, Germany). This procedure yields an at least 98% pure monocyte population, as assessed by fluorescence-activated cell sorter analysis (FACSCalibur, BD Biosciences, Franklin Lakes, NJ, USA). To obtain monocyte-derived DCs, monocytes were cultured for 5 days at 10^6^ cells/ml in RPMI 1640 medium (Gibco) containing 10% FCS in the presence of GM-CSF (50 ng/ml) and IL-4 (20 ng/ml) (both from PeproTech, Rocky Hill, NJ, USA).

### Preparation of Conditioned Media

To prepare conditioned medium from MSCs, cells at 0.2 × 10^6^ cells/ml in RPMI supplemented with 10% FCS were left untreated, stimulated for 24 h with IL-1β (25 ng/ml), IL-6 (20 ng/ml), and TNF-α (50 ng/ml) (all from PeproTech) (40) or with PBMCs at a ratio PBMC:MSC 5:1 for 5 days. In some experiments, treated MSCs were also treated with 10 µM of indomethacin (IDM) (Cayman Chemical, Ann Arbor, MI, USA). Conditioned media were obtained by centrifugation of MSCs (MSC-CM), to discard cells and debris, and different concentrations of CM were used to treat DCs.

To prepare conditioned medium from DCs (DC-CM) or DC/MSC coculture (ratio 5:1), DCs were extensively washed and cultured for further 48 h at 1 × 10^6^ cells/ml in RPMI 10% FBS without GM-CSF and IL-4. After centrifugation to discard cells and debris, the conditioned media obtained were aliquoted, stored at −20°C and used at different concentrations to induce MSC differentiation and to evaluate CCL5 production by MSCs.

### Cell Cultures

Dendritic cells cultured at 1 × 10^6^ cells/ml were stimulated for 24 h with different concentrations of prostaglandin E_2_ (PGE_2_) (10^−9^ to 10^−5^ M) (Sigma-Aldrich S.r.l. Milan, Italy), 10 µM butaprost (EP2 agonist), 10 µM misoprostol (EP2/EP3/EP4 agonist), 10 µM sulprostone (EP1/EP3 agonist) (all from Cayman Chemical), 50 µM Dioctanoyl-cAMP (d-cAMP) (Calbiochem, Merck KGaA, Darmstadt, Germania), or 50 µM forskolin (FSK) (Alexis Biochemicals, San Diego, CA, USA). In some experiments, cells at 1 × 10^6^ cells/ml were untreated or treated with different concentrations of MSC-CM previously prepared as indicated above.

Depending on the experiments, MSCs and DCs were cultured in contact or separated using transwell inserts with 0.4 µm inserts (Corning Costar, Sigma-Aldrich). MSCs, unstimulated or in the presence of IL-1β (25 ng/ml), IL-6 (20 ng/ml), and TNF-α (50 ng/ml), were plated at the bottom of 24-well plates at a seeding density of 0.05 × 10^6^ cells in 0.5 ml and DCs plated in transwell insert at 0.25 × 10^6^ cells/0.1 ml. The same ratios and volumes were used for direct contact cultures. MSCs and DCs alone were cultured and stimulated using the same number of cells/volume of the coculture. Supernatants were collected after 24 or 48 h. Where specified, DCs, MSCs, or the direct coculture of DC/MSC were treated for 48 h with 10 µg/ml Arg-Gly-Asp (RGD) (Sigma-Aldrich), 2 µg/ml cilengitide (CIL) (MedChem Express, NJ, USA), or the scrambled peptide Arg-Gly-Glu (RGE) (Sigma-Aldrich). MSCs were also pre-treated for 1 h with specific antibodies against CD44 (clone 5F12; Lifespan Biosciences, Inc., Nottingham, United Kingdom), CD29 (clone P5D2; R&D Systems, Inc., MN, USA), CD54 (clone HCD54; Biolegend, San Diego, CA, USA), and CD58 (clone TS2/9; Biolegend) or the corresponding isotype control antibody at 10 µg/ml (R&D Systems) and then cocultured with DCs (ratio DC/MSC 5:1) for 48 h.

In some experiments, MSCs alone were cultured at 0.1 × 10^6^ cells/well and treated with different concentrations of previously prepared DC-CM for 24 and 48 h.

### Flow Cytometric Analysis

Mesenchymal stromal cells were analyzed by flow cytometry with a FACSCalibur equipped with CellQuest software (BD Biosciences, Milano, Italy) using the following antibodies: anti CD44-PE and anti CD29-PE and the corresponding isotype control antibodies (all purchased from Biolegend).

### Real-Time PCR

Total MSC-RNA isolated with the Qiagen RNeasy mini kit was treated with DNase I (Qiagen, Hilden, Germany) and retrotranscribed into cDNA by using the iScript cDNA Synthesis Kit (Bio-Rad Laboratories Inc., Hercules, CA, USA). Gene specific primers were:

*Adiponectin* (ADIPOQ) (sense, 5′-AGGGTGAGAAAGGAGATCC-3′; antisense, 5′-GGCATGTTGGGGATAGTAA-3′), *FABP4* (sense, 5′-TGGTTGATTTTCCATCCCAT-3′; antisense, 5′-TACTGGGCCAGGAATTTGAC-3′), *PPARγ* (sense, 5′-CCTATTGACCCAGAAAGCGATT-3′; antisense, 5′-CATTACGGAGAGATCCACGGA-3′), alkaline phosphatase (*ALP*) (sense, 5′-AGCACTCCCACTTCATCTGGAA-3′; antisense, 5′-GAGACCCAATAGGTAGTCCACATTG-3′), *RUNX2* (sense, 5′-AGAAGGCACAGACAGAAGCTTGA-3′; antisense, 5′-AGGAATGCGCCCTAAATCACT-3′), *CCL5* (sense, 5′-CCTCATTGCTACTGCCCTCT-3′; antisense, 5′-ACGACTGCTGGGTTGGAGCACTT-3′), *RPL13A* (sense, 5′-CATAGGAAGCTGGGAGCAAG-3′; antisense, 5′-GCCCTCCAATCAGTCTTCTG-3′). The iQ™ SYBR Green Supermix (Bio-Rad Laboratories Inc., Segrate, MI, Italy) for quantitative real-time PCR was used according to manufacturer instructions. Reactions were run in duplicate on an iCycler Chromo4™ (Bio-Rad Laboratories Inc.) and Opticon Monitor™ 3.0 Software and Genex Macro were used for data analysis (Bio-Rad Laboratories Inc.). Gene expression was normalized based on RPL13A mRNA content.

### ELISA

Cell-free supernatants were harvested and OPN and CCL5 production was measured by ELISA assay (R&D Systems, Minneapolis, MN, USA). PGE_2_ production was assessed by EIA kit (Cayman Chemical).

### Adipogenic Induction

Mesenchymal stromal cells were cultured with DMEM and passaged twice/three times. Then, cells were seeded into 12-well plates, and adipogenic induction was performed using StemMACS™ AdipoDiff Media (Miltenyi Biotec). Cells were cultured in presence of complete adipogenic medium or with 70% AdipoDiff Media plus 30% DC-CM or DC/MSC-CM or 30% basal medium, or recombinant human OPN (1 µg/ml) (Peprotech). Medium was changed every 4/5 days and mRNA extraction was performed at 5 and 12 days while lipid droplet staining was evaluated at 15 days of culture. In some experiments, cells cultured in presence of DC-CM were treated with neutralizing monoclonal antibodies against CD44 (clone 5F12; Lifespan Biosciences, Inc.) and CD29 (clone P5D2; R&D Systems) or with the corresponding isotype control antibody at 10 µg/ml (R&D Systems).

### Osteogenic Induction

Mesenchymal stromal cells were seeded into 12-well plates, and osteogenic induction was performed using DMEM medium supplemented with 50 µM ascorbic acid, 10 mM beta glycerophosphate, and 100 nM dexamethasone (all from Sigma-Aldrich). MSCs were cultured in presence of complete osteogenic medium or with 70% osteogenic medium plus 30% DC-CM or 30% basal medium, or recombinant human OPN (1 µg/ml). mRNA extraction was performed at 7 and 14 days and Alizarin staining at 14 and 21 days.

### Oil Red O Staining

To evaluate adipogenesis, cells were fixed in 4% paraformaldehyde for 10 min at RT, washed twice with distilled water, and incubated with 60% isopropanol for 10 min at RT. Then, solution was removed and cells were incubated in fresh Oil Red O (1.8 in 60% isopropanol) (Sigma-Aldrich) for 5 min at RT. Cells were washed with isopropanol, and induced cells were visible as cells containing consistent red deposits in vacuoles. Positive cells were visualized by light microscopy and photographed and the percentage of differentiated cells was determined by counting cells based on Oil Red O staining in the lipid vacuoles (adipocytes were counted in five random fields). Quantification of lipid accumulation is achieved by Oil Red O extraction by lysis (100% isopropanol) and gentle agitation for 10 min at room temperature. Following Oil Red O extraction, 150 µl are transferred to a 96-well plate and absorbance measured at 490 nm using a plate reader.

### Alizarin Red S Staining

The culture medium was discarded, and the cells were gently rinsed with PBS twice. Then, the cells were fixed with 4% paraformaldehyde for 10 min at room temperature. The cells were washed with distilled water three times and stained with 2% Alizarin red S (Sigma-Aldrich) for 30 min at RT. The cells were washed again with distilled water three times. Finally, the cells were rinsed with water, and the Alizarin red S staining was observed using a microscope. The formation of red calcium deposits is a marker of osteogenic differentiation. To quantify Alizarin Red S staining, the stained cells were incubated in 10% acetic acid for 30 min and absorbance was measured at 405 nm with a microplate reader.

### Immunohistochemistry

In order to validate our hypothesis *ex vivo*, we selected thirteen BM trephine biopsy (BMB) from the Pathology archive of the University of Brescia. Ten normal BMB were selected from staging biopsy, negative for lymphoma; additionally, we selected three cases with diagnosis of myelofibrosis, a pathological condition known to be associated with increased expression of CD271/NGFR (41).

Immunohistochemistry was performed on 2 µm sections of formalin fixed-paraffin embedded tissue using the following antibodies: OPN (Polyclonal Goat IgG, R&D Systems, dilution, 1:65), CD1c (clone OTI2F4, Abcam, Cambridge, United Kingdom, dilution 1:300), CD14 (clone 7, Leica Microsystems, Wetzlar, Germany, dilution 1:50), CD38 (clone SPC32, Leica Microsystems, dilution 1:100), CD271/NGFR (clone 7F10, Leica Microsystems, kindly provided by Prof Tripodo, University of Palermo, Italy, dilution 1:50), E-cadherin (clone 36, Ventana Medical Systems, Tucson, AZ, USA, prediluted), Myeloperoxidase (polyclonal, Dako-Agilent Technologies Santa Clara, CA, USA, dilution 1:6,000). Double and triple stainings were performed as previously described (42).

### Statistical Analysis

Statistical significance was determined by using non-parametric Student’s *t-*test and one-way analysis of variance, as appropriate. Results were analyzed by using GraphPad PRISM 5.0 software.

## Results

### The Activation State of MSCs Dictates OPN Production in DC/MSC Cocultures

**Figure 1:**
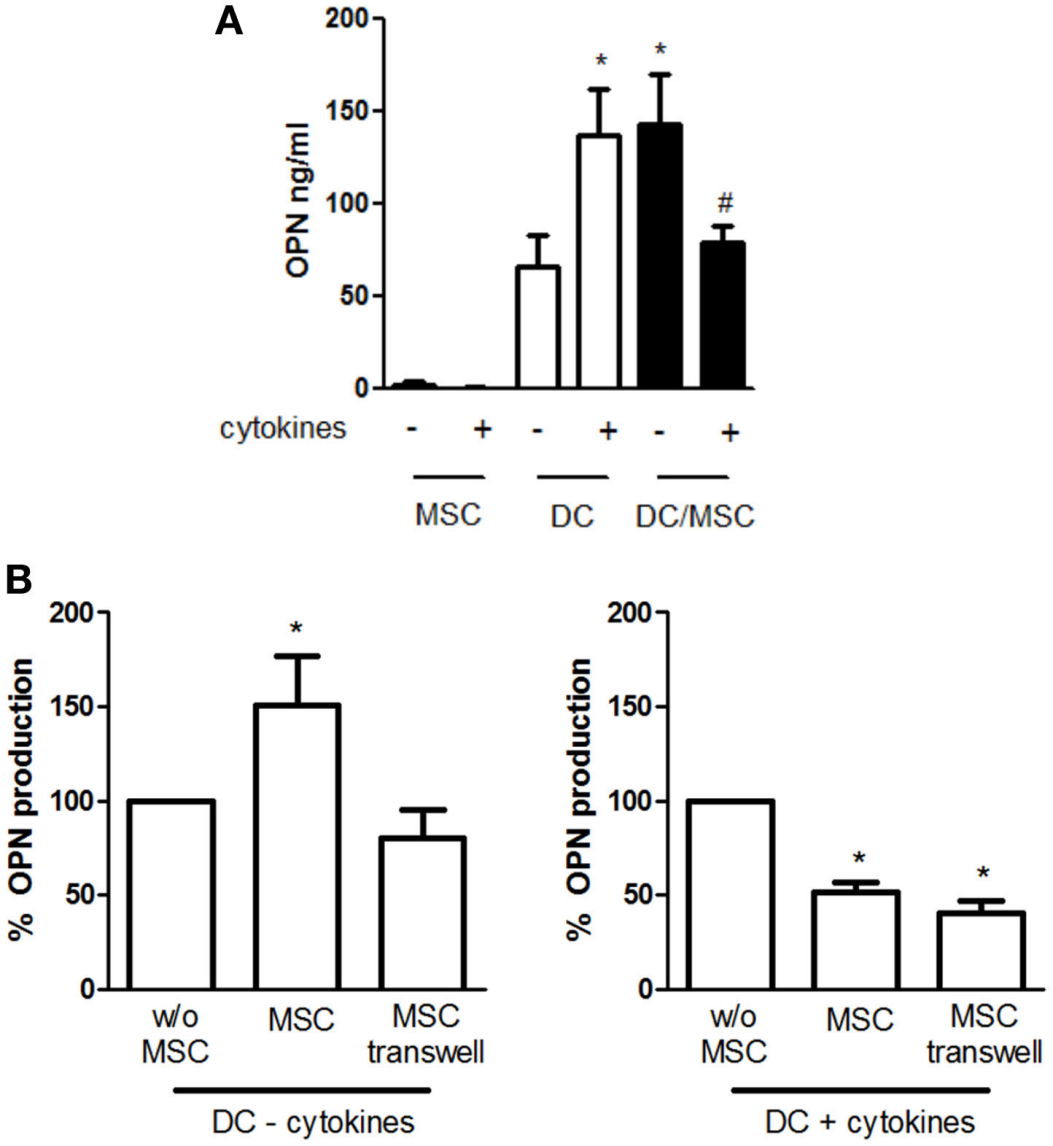
Influence of mesenchymal stromal cells (MSCs) on osteopontin (OPN) production by dendritic cells (DCs). **(A)** DCs were cultured alone or with MSCs (ratio DC/MSC 5:1) in the absence or in the presence of the proinflammatory cytokines (IL-1β, TNF-α, IL-6) for 24 h and OPN levels was determined in the supernatants by ELISA. Results are expressed as mean ± SEM of 10 independent experiments. *p < 0.05 vs. DCs alone in resting conditions ^#^*p* < 0.05 vs. DC/MSC coculture in resting conditions and vs. DCs alone in presence of cytokines by Student’s *t*-test. **(B)** DCs were cultured without MSCs or with MSCs in contact or in transwell systems for 24 h in resting conditions (left panel) or in presence of proinflammatory cytokines (right panel). Tested ratio were DC/MSC 5:1. OPN levels were measured by ELISA and expressed as % of OPN production by DCs alone set as 100% (mean ± SEM of five independent experiments). *p < 0.05 vs. DCs alone by Student’s *t*-test.

We and others have previously shown that high levels of OPN are released by DCs in both resting and stimulated conditions. To investigate the influence of adipose tissue-derived MSCs on OPN production by DCs, cells were cocultured in absence or in the concomitant presence of TNF-α, IL-6, and IL-1β. This previously used cocktail (40) is known to activate MSCs, better than each cytokine alone (43). As shown in Figure 1A, DCs constitutively produce high levels of OPN that were further increased in the presence of proinflammatory cytokines (66 ± 16.9 vs. 136.6 ± 25.4 ng/ml OPN with resting and stimulated DCs, respectively). On the contrary, resting MSCs released low amounts of OPN, as previously reported (44), and no differences were observed in activated conditions. However, when DCs were cocultured with MSCs (5:1 ratio) in the absence of deliberate stimulation, the production of OPN strongly increased (142.5 ± 25 ng/ml). Conversely, in the presence of proinflammatory cytokines, OPN levels in the cocultures (79 ± 8.9 ng/ml) were similar to those obtained in DC cultures alone. Different ratios of DCs and MSCs derived from adipose tissue or BM were tested and the maximal effect was observed at the DC/MSC ratio of 5:1 independently of the origin of MSCs (not shown); therefore, the DC/MSC 5:1 ratio with MSCs purified from adipose tissue was selected for further studies.

To investigate the mechanisms responsible for MSC-mediated regulation of OPN production, DCs and MSCs were cultured either in contact or in transwell conditions. As depicted in Figure 1B, the OPN upregulation was prevented when unstimulated MSCs and DCs were physically separated, implicating a prominent role of cell–cell contact in the process. In contrast, OPN suppression remained fully evident in the transwell co-cultures of activated MSCs and DCs, suggesting that inhibition was mediated by soluble factors.

### Mechanisms Involved in the Regulation of OPN Production in DC/MSC Cocultures

**Figure 2:**
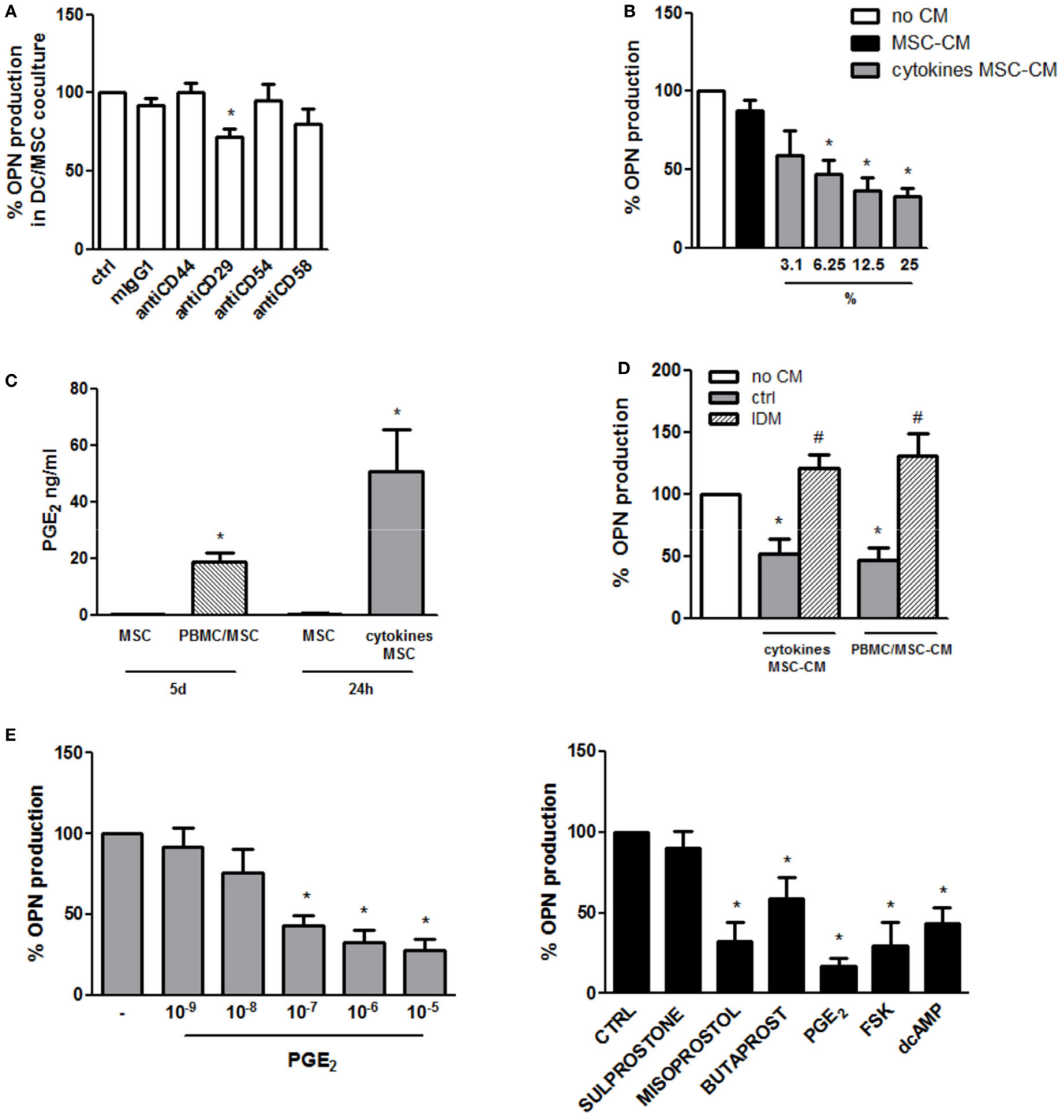
Mechanisms involved in the regulation of osteopontin (OPN) production in DC/mesenchymal stromal cells (MSC) cocultures. **(A)** MSCs were pretreated for 1 h with the specific neutralizing antibodies against CD44, CD29, CD54, and CD58 or the corresponding isotype ctrl (mIgG1) and then cultured with dendritic cells (DCs) (ratio DC/MSC 5:1) for 48 h. Supernatants were collected and tested for OPN production by ELISA. OPN levels are expressed as % of OPN production in DC/MSC alone set as 100% (mean ± SEM of four independent experiments). *p < 0.05 vs. DC/MSC coculture untreated by Student’s *t*-test. **(B)** DCs were cultured alone or with the addition of conditioned medium from MSCs unstimulated (25% v/v) or with different concentrations of conditioned medium from MSCs activated for 24 h with proinflammatory cytokines (MSC-CM or cytokines MSC-CM). Supernatants were collected after 24 h and tested for OPN production by ELISA and expressed as % of OPN production by DCs alone set as 100%. *p < 0.05 vs. DCs alone by one-way ANOVA followed by Tukey’s Multiple Comparison Test. **(C)** PGE_2_ levels were measured by ELISA in supernatants from MSCs unstimulated or cocultured for 5 days with PBMCs (PBMC/MSC) or untreated or treated for 24 h with proinflammatory cytokines (cytokines MSC). Results are representative of four and three experiments, respectively. *p < 0.05 vs. MSC unstimulated by Student’s *t*-test. **(D)** DCs were cultured alone (no CM) or in the presence of supernatants from MSCs stimulated with proinflammatory cytokines for 24 h (cytokines MSC-CM) or co-cultured with PBMC (ratio PBMC/MSC 5:1) for 5 days (PBMC/MSC-CM). MSC stimulation was performed with or without indomethacin (IDM). After 24 h, DC supernatants were collected and tested for OPN production by ELISA. Results are expressed as % of OPN production by DCs alone set as 100% (mean ± SEM of six independent experiments). *p < 0.05 vs. DCs alone (no CM); ^#^*p* < 0.05 vs. MSC-CM without IDM (ctrl) by one-way ANOVA followed by Tukey’s Multiple Comparison Test. **(E)** DCs were stimulated with different concentrations of PGE_2_. Supernatants were harvested 24 h later and subjected to OPN ELISA (left panel) (mean ± SEM of six independent experiments). DCs were stimulated with Sulprostone (EP1/3 agonist), Butaprost (EP2 agonist), Misoprostol (EP2/3/4 agonist), PGE_2_, Forskolin (FSK), and dcAMP. After 24 h, supernatants were collected and tested for OPN by ELISA (right panel) (mean ± SEM of four independent experiments). Results are expressed as % of OPN production by DCs alone set as 100%; *p < 0.05 vs. DCs alone by one-way ANOVA followed by Dunnett’s Multiple Comparison Test.

Given the necessity for DC/MSC contact for the induction of OPN in resting conditions, the requirement of adhesion molecules was investigated. Blocking of CD44, CD54, and CD58 by MSC pretreatment with neutralizing antibodies did not affect OPN production. On the contrary, a moderate but statistically significant reduction of OPN levels was observed blocking CD29 (Figure 2A).

In order to investigate the influence of soluble factors in OPN inhibition in stimulated conditions, DCs were treated with conditioned medium of MSCs unstimulated or activated with TNF-α, IL-6, and IL-1β. Conditioned medium of activated MSCs, but not of control MSCs, was able to reduce in a dose-dependent manner the production of OPN by DCs (Figure 2B). Because of the documented immunosuppressive role of PGE_2_ produced by MSCs (22, 45), the possible involvement of this eicosanoid was investigated. Figure 2C shows that, as previously reported (46, 47), also in our culture conditions high levels of PGE_2_ are produced by MSCs activated with proinflammatory cytokines or cocultured with PBMCs. Consistently with this result, conditioned medium from MSCs activated in the presence of IDM, an inhibitor of cyclooxigenase-1, failed to reduce OPN in DCs (Figure 2D). Furthermore, treatment of DCs with exogenous PGE_2_ (10^−9^ to 10^−5^ M) led to a dose-dependent inhibition of OPN production (Figure 2E, left panel). Figure 2E (right panel) shows that by the use of receptor antagonists EP2 and EP4 were identified as the receptors responsible for PGE_2_-mediated OPN production. Consistent with previous finding that the EP2/EP4 activation pathway is coupled with an increase in cAMP production, both d-cAMP and FSK suppressed OPN expression in DCs (Figure 2E, right panel).

### OPN Secreted by DCs Regulates Adipogenic and Osteogenic Differentiation of MSCs

**Figure 3:**
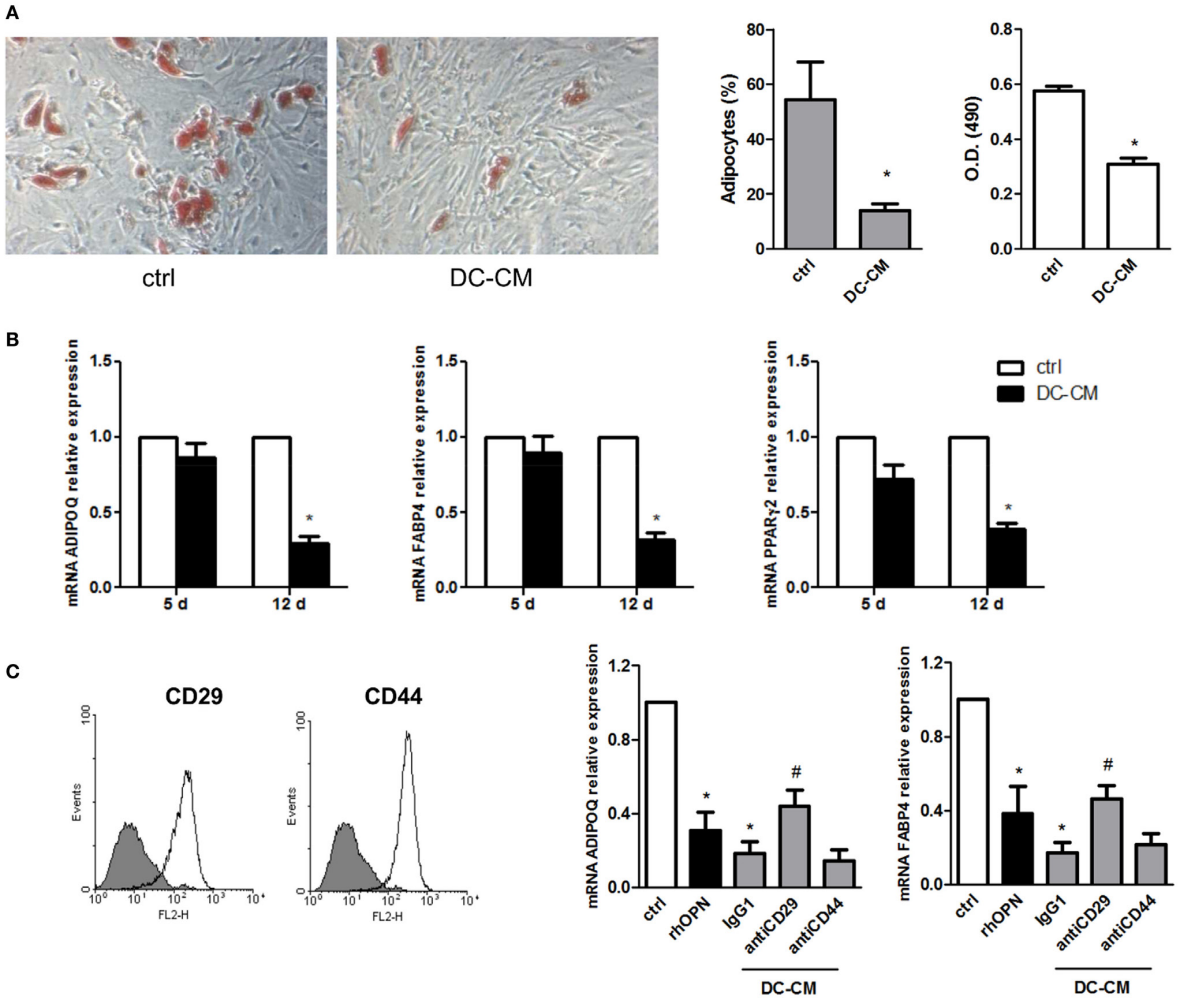
DC-conditioned medium inhibits mesenchymal stromal cells (MSC) differentiation into adipocytes through osteopontin release. **(A)** MSCs were cultured for 15 days in adipocyte differentiation medium in the presence of 30% DC-CM or RPMI (control condition, ctrl) and stained with Oil Red O to reveal lipid droplets (original magnification 5×) (left panel). Adipocytes were counted in five random fields from one representative well per group (middle panel) and Oil Red O extracted with isopropanol was measured at optical density 490 (right panel) (mean ± SEM of four independent wells). *p < 0.05 vs. ctrl by Student’s *t*-test. **(B)** The mRNA levels of *ADIPOQ, FABP4*, and *PPAR*γ*2* were analyzed by real-time PCR at days 5 and 12 of culture. Data were shown as means ± SEM (*n* = 3). *p < 0.05 vs. ctrl by Student’s *t*-test. **(C)** MSCs were examined for the expression of CD29 and CD44 by flow cytometry (gray area, isotype control; white area, specific antibody). MSCs were induced by adipogenic differentiation medium in control condition, with rhOPN or DC-CM in the presence or the absence of the indicated antibodies. Relative mRNA expression of ADIPOQ and FABP4 was measured by real-time PCR on day 12 of adipogenic induction. *RPL13A* was used for normalization. Data were shown as means ± SEM (*n* = 3). *p < 0.05 vs. ctrl; ^#^*p* < 0.05 vs DC-CM in presence of the isotype control by one-way ANOVA followed by Tukey’s Multiple Comparison Test.

The possible functional effect of OPN regulation observed in MSC/DC cocultures on MSC differentiation was thus investigated. First, MSCs were induced to differentiate into adipocytes in the presence of resting DC-derived conditioned medium (DC-CM). Analysis of lipid droplet production detected by Oil Red O showed fewer lipid droplets in the cytoplasm of adipocytes treated with DC-CM for 15 days, compared to control cells. By microscopic evaluation, the percentage of adipocytes was 14 ± 2.5 vs. 54.4 ± 13.6% in presence of DC-CM or control medium, respectively. Compared to DC-CM, DC/MSC-CM did not differ significantly in the adipogenesis inhibition (the adipocytes percentage was 15.3 ± 1.7% with DC-CM vs. 12.4 ± 2.0% with DC/MSC-CM in two independent experiments). The results obtained with DC-CM were further supported by quantification of the solubilized stain, with 1.8-fold lower absorbance values with DC-CM vs. control-differentiated MSCs (Figure 3A). In agreement with this result, it was observed in MSCs differentiated in presence of DC-CM, a reduction in the expression of markers normally associated with adipogenesis, such as adiponectin (*ADIPOQ*), *FABP4*, and *PPARγ*, assessed by RT-PCR at 12 days of culture (Figure 3B). Since MSCs express CD29 and CD44, two receptors involved in OPN signaling (Figure 3C, left panel), MSCs were induced to differentiate into adipocytes in presence of human recombinant OPN (rhOPN) or of DC-CM in the presence of the neutralizing antibodies for CD29 and CD44. Figure 3C (right panel) shows that, as expected, recombinant OPN and DC-CM reduced the expression of the adipose-specific genes *ADIPOQ* and *FABP4* and that blocking of integrin β1 (antiCD29 moAb) reverted the inhibitory effect of DC-CM. No involvement of CD44 was apparently observed.

**Figure 4:**
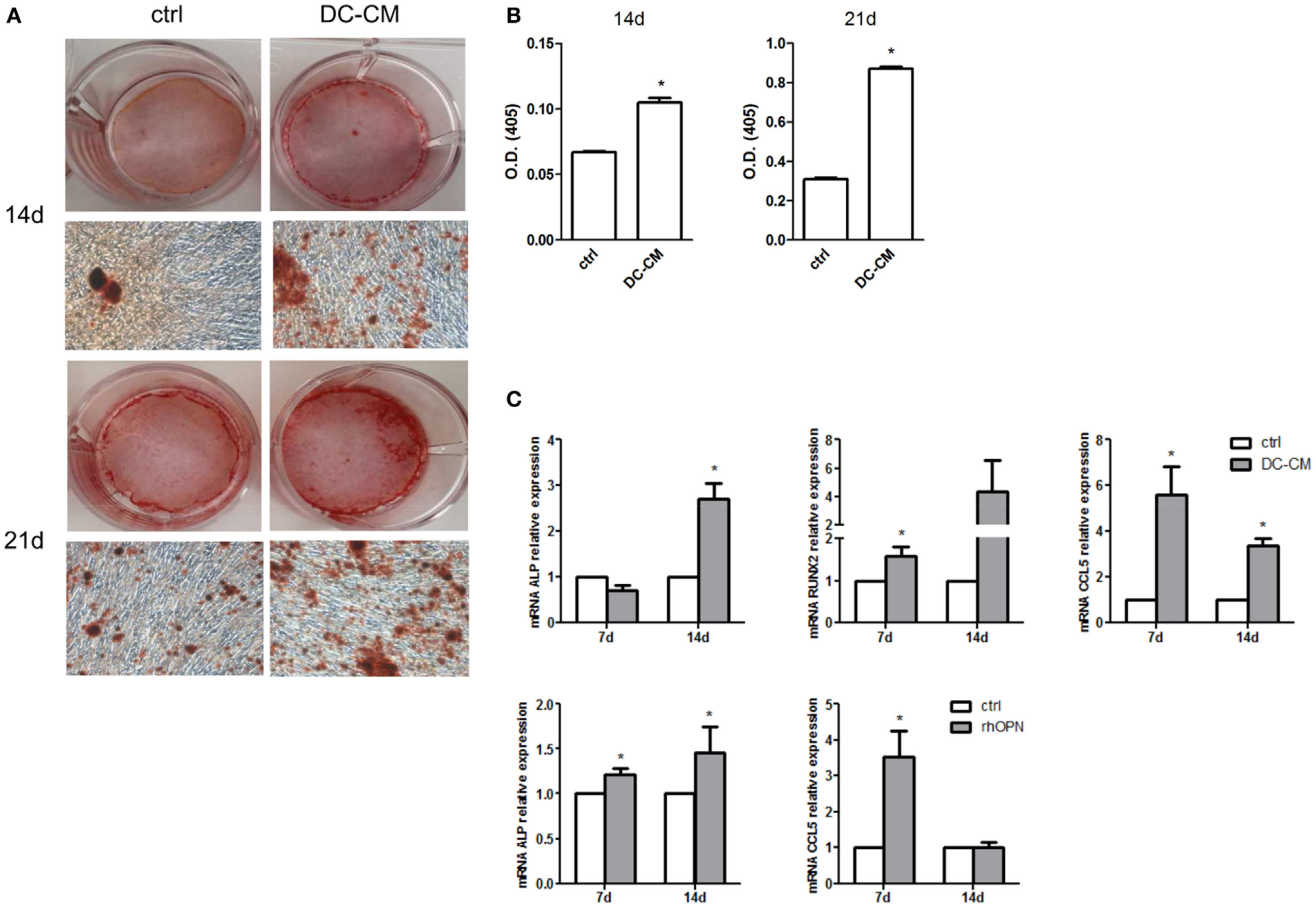
DC-conditioned medium induces mesenchymal stromal cells (MSC) differentiation into osteoblasts through osteopontin release. **(A)** MSCs were cultured for 14 and 21 days in osteogenic differentiation medium in the presence of 30% DC-CM or RPMI (control condition, ctrl) and stained with Alizarin Red S to identify mineralized deposits. **(B)** Quantification of Alizarin Red staining via dissolving the dye and measurement of subsequent absorption at optical density of 405 nm. *p < 0.05 vs. ctrl by Student’s *t*-test. **(C)** The mRNA levels of *alkaline phosphatase, RUNX*, and *CCL5* were analyzed by real-time PCR at days 7 and 14 of culture of MSC in presence of DC-CM or rhOPN. *RPL13A* was used for normalization. Data were shown as means ± SEM (*n* = 3). *p < 0.05 vs. ctrl by Student’s *t*-test.

In parallel, MSCs were also induced to differentiate into osteoblasts in the presence of DC-CM. As shown in Figure 4A, a strong intensity staining with Alizarin Red S could be detected at 14 and 21 days in presence of DC-CM compared to control medium. Alizarin Red stain quantification confirmed that in presence of DC-CM osteoblast differentiation was approximately 1.6- and 2.8-fold higher than in control cells at 14- and 21-day culture, respectively (Figure 4B). As expected based on these results, the mRNA expression of osteo-specific genes, such as the *ALP* and Runt-related transcription factor 2(*RUNX2*), were increased in cells cultured in the presence of DC-CM or rhOPN at 7 and 14 days. Moreover, the expression of CCL5, a chemokine recently shown to be involved in the induction of osteogenesis of MSCs (48) was also significantly higher in MSCs differentiated with DC-CM (at 7 and 14 days) or with rhOPN (at 7 days) compared to control cells (Figure 4C). Taken together, our results strongly suggest that OPN secreted from DCs skew MSC differentiation to osteogenesis.

### OPN Regulates CCL5 Production in the DC/MSC Cocoultures

**Figure 5:**
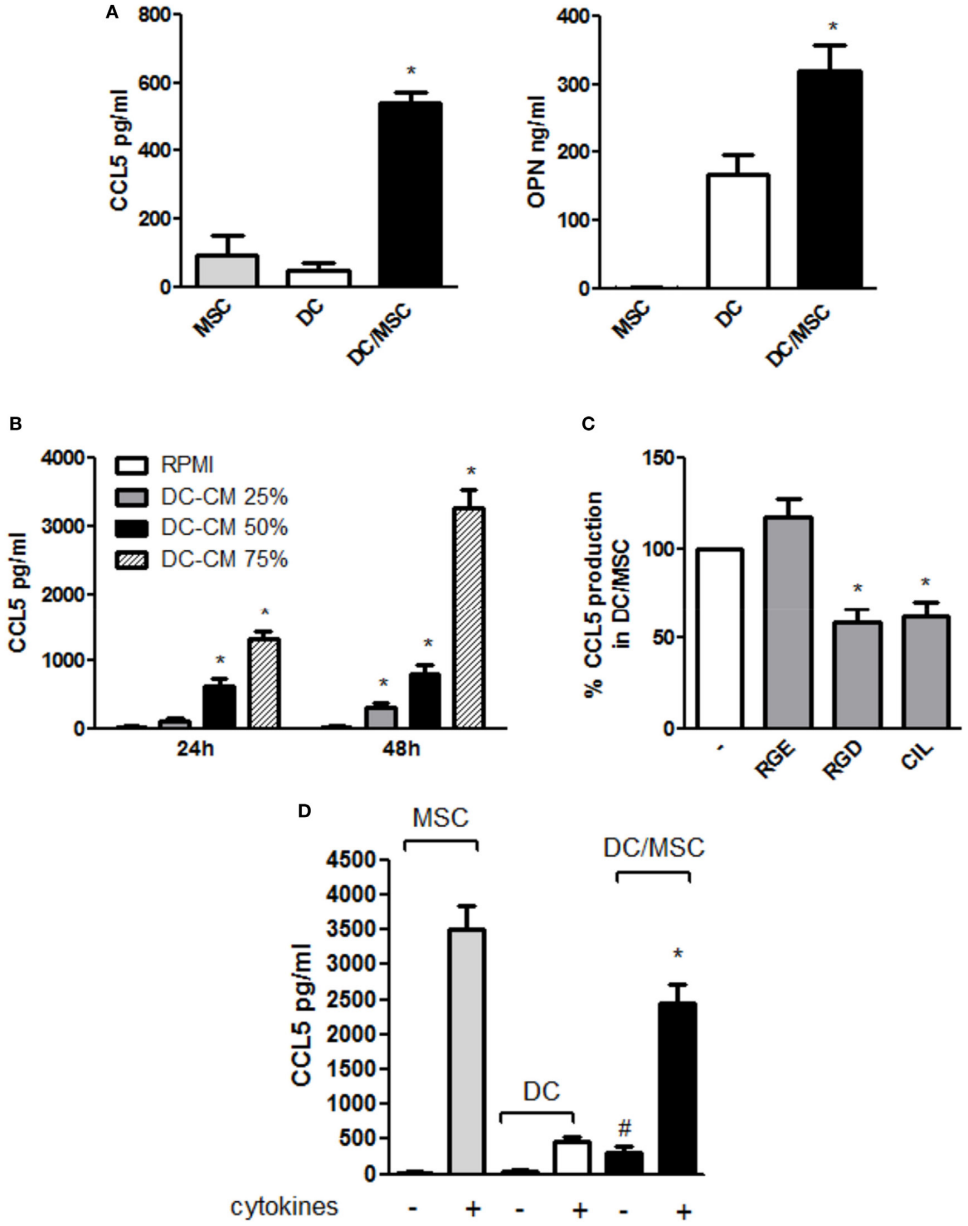
Osteopontin (OPN) released by dendritic cells (DCs) modulates CCL5 production in DC/mesenchymal stromal cells (MSC) coculture. **(A)** DCs were cultured in the absence or in the presence of MSCs. Supernatants were harvested after 48 h and assayed by ELISA for CCL5 (left) and OPN (right) (mean ± SEM of four independent experiments). *p < 0.05 vs. DC alone by one-way ANOVA followed by Tukey’s Multiple Comparison Test. **(B)** MSC were incubated for 24 and 48 h with different concentrations of DC conditioned medium (DC-CM) and CCL5 concentrations were measured in the supernatants by ELISA (mean ± SEM of four independent experiments). *p < 0.05 vs. MSC alone at 24 and 48 h, respectively by Student’s *t*-test. **(C)** DCs and MSCs were cocultured for 48 h in the presence or in the absence of the antagonistic integrin inhibitors RGD (10 µg/ml) and cilengitide (CIL) (2 µg/ml). RGE was used as a control peptide. CCL5 concentrations were tested by ELISA. Results are expressed as % of CCL5 production by DC/MSC set as 100% and are representative of four independent experiments. *p < 0.05 vs. DC/MSC coculture in presence of RGE by Student’s *t*-test. **(D)** DCs were cultured alone or with MSCs (ratio DC/MSC 5:1) in the absence or in the presence of the proinflammatory cytokines (IL-1β, TNF-α, IL-6) for 48 h and OPN levels was determined in the supernatants by ELISA (mean ± SEM of seven different experiments). ^#^*p* < 0.05 vs. DCs alone in the absence of cytokines; *p < 0.05 vs. MSCs alone in the presence of cytokines by Student’s *t*-test.

CCL5 upregulation observed during osteogenesis in the presence of DC conditioned medium prompted us to further explore CCL5 production in DC-MSC crosstalk. As shown in Figure 5A (left panel), CCL5 levels in the DC/MSC coculture were significantly higher compared to DCs and MSCs cultured alone (about sixfold increase with respect to MSCs alone and about 10-fold increase with respect to DCs alone), similarly to what observed for the production of OPN (Figure 5A, right panel). To demonstrate a possible role of OPN in CCL5 production, MSCs were stimulated with DC-CM and CCL5 was quantified. In the presence of DC-CM, CCL5 levels augmented in a concentration dependent manner at 24 and 48 h stimulation with the peak attained at 48 h (Figure 5B). Moreover, inhibition of RGD integrins, a family of OPN receptors, with two different integrin antagonists Arg-Gly-Asp (RGD) and Cilengitide (CIL), significantly reduced CCL5 expression. As expected, the control peptide Arg-Gly-Glu (RGE) did not alter CCL5 production (Figure 5C). Taking together, these results suggest that, in resting conditions, OPN released by DCs induces CCL5 upregulation. CCL5 production highly differed between resting and inflammatory conditions. As shown in Figure 5D, in the presence of proinflammatory cytokines, high levels of CCL5 were detected in DCs, and even more in MSCs, cultured alone. In DC/MSC coculture, CCL5 levels are conspicuous, though lower than those produced by MSCs alone and lower than the sum of the values for each cell type cultured separately.

### Interaction of CD1c^+^ DCs and MSCs in Human BM

**Figure 6:**
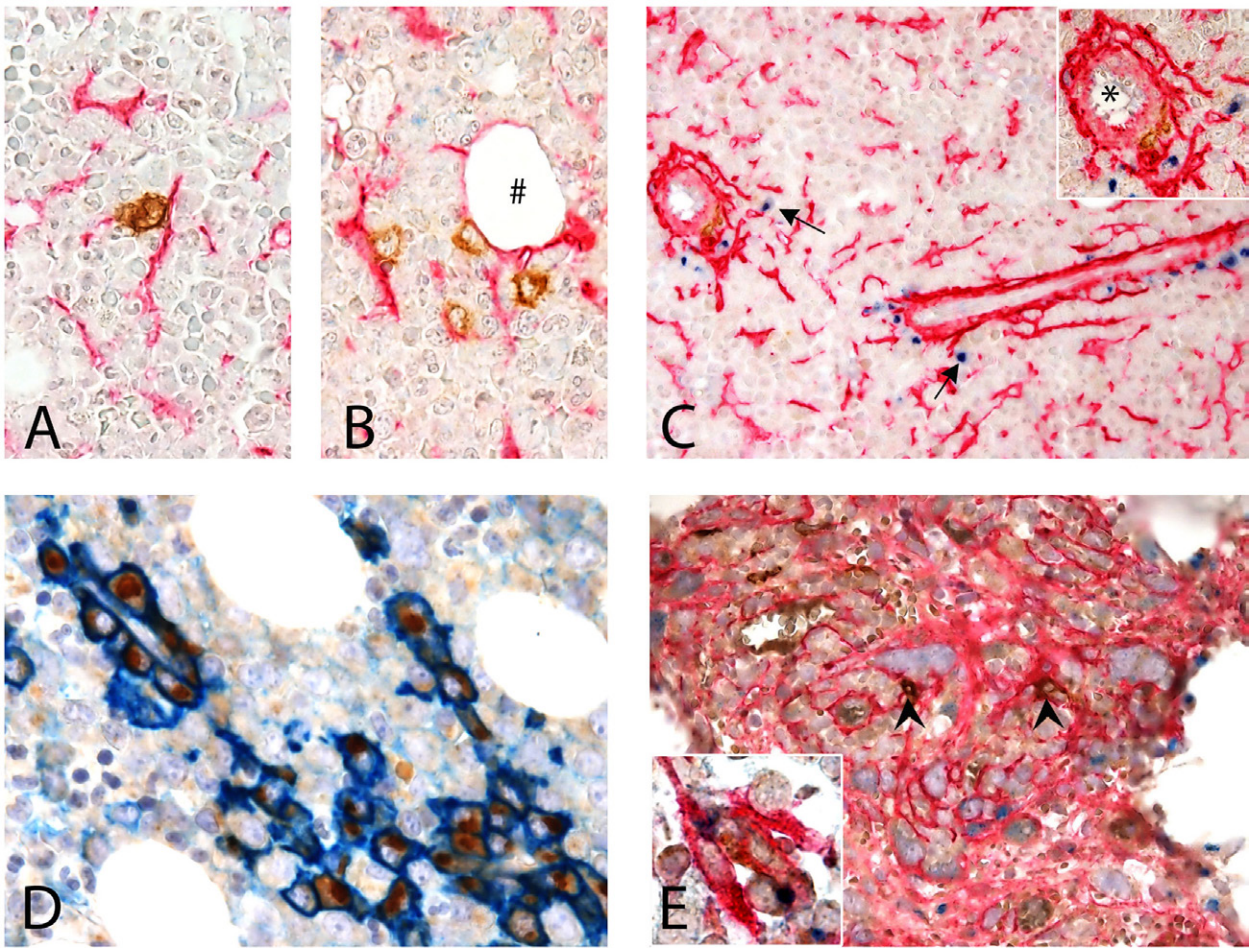
Interaction of CD1c + dendritic cells (DCs) and CD271 + mesenchymal stromal cells (MSCs) in human BM. **(A–D)** Normal BM. **(A,B)** Show the interaction of CD1c^+^ (brown) DCs with CD271/NGFR^+^ (red) MSCs in the interstitium of normal hematopoietic matrix, even near an adipocyte (#). **(C)** Shows this interaction next to a vessel (*) in the proximity of which osteopontin (OPN) (blu) is produced (arrows). Double immunostaining for CD38 (blue) and OPN (brown) showed that plasma cells are the major expressors of OPN in normal human BM **(D)**. **(E)** BM with myelofibrosis: intertrabecular spaces show dense network of CD271^+^ MSCs (red) that interact with scattered CD1c^+^ DCs (brown, arrow heads); in this contest, OPN (blue) production can be detected [**(E)**, inset].

CD1c^+^ cells in normal human BM are rare and display a round shape with no dendrites. CD1c^+^ cells may be interstitial (Figures 6A,B) or perivascular (Figure 6C) and, as shown by double immunostaining, they do interact with CD271^+^ MSCs. This observation confirms what originally suggested by Dokić et al. (23) in a different setting and fully supports our hypothesis.

When evaluating OPN expression in normal human bone marrow biopsies (BMB), we found granular positivity in the cytoplasm of megakaryocytes, a distinctive Golgian-dot expression in the cytoplasm of cells lining small capillaries and in scattered interstitial cells (Figure 6C). No evidence of interstitial OPN, previously described in plasma cell neoplasms (49), was detected in normal BMB.

By double immunostaining, OPN^+^ cells were negative for Myeloperoxidase and E-cadherin, excluding respectively myeloid and erythroid precursors; CD14^+^ monocytes were also negative (not shown). The large majority of OPN^+^ cells were CD38^+^ plasma cells, lined up in perivascular spaces, suggesting that such expression is constitutive in reactive plasma cells and not exclusive of pathological plasma cell proliferations (49) (Figure 6D). When immunostaining normal BMB with CD1c and OPN, only weak OPN expression was observed in proximity of rare CD1c^+^ DC while in myelofibrotic BMB OPN^+^ DC could be clearly found in the contest of DC–MSC interaction (Figure 6E, inset). Notably, this difference may be explained by the strong increase of CD271^+^ stromal stem cells in this pathological condition (Figure 6E). Although these findings on human tissue need to be confirmed in a larger cohort, they support our *in vitro* observations.

## Discussion

Mesenchymal stromal cells are known to control immune cell functions and to regulate immune responses in both homeostatic and inflammatory conditions. Our results from *in vitro* studies show that OPN production in DC/MSC cocultures is regulated by the state of activation of MSCs and that OPN released by DCs can influence MSC differentiation. Moreover, we detected OPN production in the contest of MSC-DC colocalization in tissue sections of human BM.

Dendritic cells are known to be an important source of OPN both *in vitro* and *in vivo* (28, 34), and this study shows that resting MSCs can further increase OPN production when cocultured with DCs. On the contrary, in the presence of proinflammatory cytokines, MSCs exert an opposite effect inhibiting OPN production. Coculture experiments of MSCs and DCs performed with transwell inserts indicated that MSCs regulate OPN production both by the release of soluble factors and by a cell–cell contact mechanism. MSCs constitutively express adhesion molecules that are involved in the direct interaction between MSCs and immune cells (50). For example, CD58 and CD54 play an important role in MSC/T cells interaction (50). Similarly, CD44 and CD29 are involved in the contact of human hematopoietic progenitor cells and MSCs (51, 52). By the use of blocking monoclonal antibodies, it was possible to show that resting MSCs upregulate OPN production by a cell–cell contact mechanism involving the adhesion molecule CD29; other membrane proteins, such as CD44, CD54, and CD58, were not apparently involved in OPN production. However, the partial reduction of OPN observed using anti-CD29 indicates that other interactions likely contribute to this process.

Conversely, the inhibition of OPN production by activated MSCs was mediated by the release of soluble factors. PGE_2_ released by MSCs was identified as the main soluble factor responsible for this inhibition. This conclusion is supported by multiple experimental approaches, including the use of IDM to block PGE_2_ production, the pharmacological blocking of the two main PGE_2_ receptors expressed by DCs, namely EP2 and EP4, and the increasing of intracellular concentration of cyclic AMP, the main second messenger downstream EP2/EP4 activation (53–55).

Although in the past few years, the attention was mostly focused on the ability of MSCs to regulate immune cells, such as DCs, it is conceivable that cell-to-cell communication is bidirectional, with DCs also being able to influence MSC functions (56). The influence of immune cells on MSCs lineage commitment is an intriguingly field that has increasingly attracted great attention in recent years (19). In this line, lymphocytes were reported to inhibit MSC-driven bone regeneration through the secretion of IFN-γ and TNF-α (57). Similarly, mononuclear phagocytes were reported to drive osteogenic differentiation (58, 59). No evidence of a direct role of DCs in MSCs differentiation is so far available. To our knowledge, there is only a report by Pamir et al. (60) indicating that under physiological conditions DCs play a role in adipose tissue homeostasis.

Here, we report that medium conditioned by resting DCs inhibits MSC differentiation to adipocytes by a mechanism that depends on the presence of OPN. The capacity of OPN to interfere with adipocyte development and functions is also substantiated by the findings that OPN deficiency in mice promotes adipogenesis (61).

While inhibiting adipogenesis, DC supernatant promoted MSC differentiation to osteoblasts, as revealed by the increased Alizarin Red S staining. This dual opposite regulation was paralleled by the dowregulation of genes involved in adipogenesis (e.g., *ADIPOQ, FAB4*, and *PPARγ2*) and in the upregulation of genes involved in osteogenesis, such as *ALP, RUNX2*, and *CCL5*. In this regard, it is interesting to note that CCL5 and its receptor CCR1 were shown to be required for osteogenesis of human MSCs (48, 62). In the cocultures MSC/DC, characterized by high levels of OPN, CCL5 expression was increased compared with DCs and MSCs alone, suggesting that OPN might contribute to CCL5 induction. Indeed, our studies using supernatants from DCs and RGD peptide demonstrate that DC-derived OPN induces CCL5 production from MSCs. Thus, we propose that OPN might induce osteogenic differentiation through a direct effect and an amplification loop *via* CCL5. Interestingly, in line with our results, tumor-derived OPN has been shown to induce MSC expression of CCL5 to enhance tumor growth and metastasis (63). Altogether, these results support the existence in MSC–DC cocultures of a pro-osteogenic cytokine network orchestrated by the production of OPN by DCs.

An important question to be answered is where the interaction between DCs and MSCs might take place. Localization studies identified MSCs in the perivascular regions of many tissues (healthy adipose tissue, BM niche, periapical lesion) and direct DC–MSC interaction was shown to happen *in situ* in periapical lesions (23). In this study, we show for the first time the co-localization of CD1c^+^ DCs and CD271^+^ MSCs in human BM from control subjects and patients with myelofibrosis, a pathology characterized by high CD271 expression. As previously reported (64), human BM specimen demonstrated few (estimated 1% or less) CD1c^+^ DCs; however, double immunostaining revealed a remarkably intimate relationship between CD271^+^ MSCs and CD1c^+^ cells. In BM, the large majority of OPN^+^ cells were CD38^+^ plasma cells lining small capillaries spaces. By double immunostaining with OPN and CD1c, OPN^+^ DCs were found in BM in the contest of DC–MSC interaction. Further studies are required to better elucidate the role of OPN in the interplay between DCs and MSCs in normal and pathological conditions.

**Figure 7:**
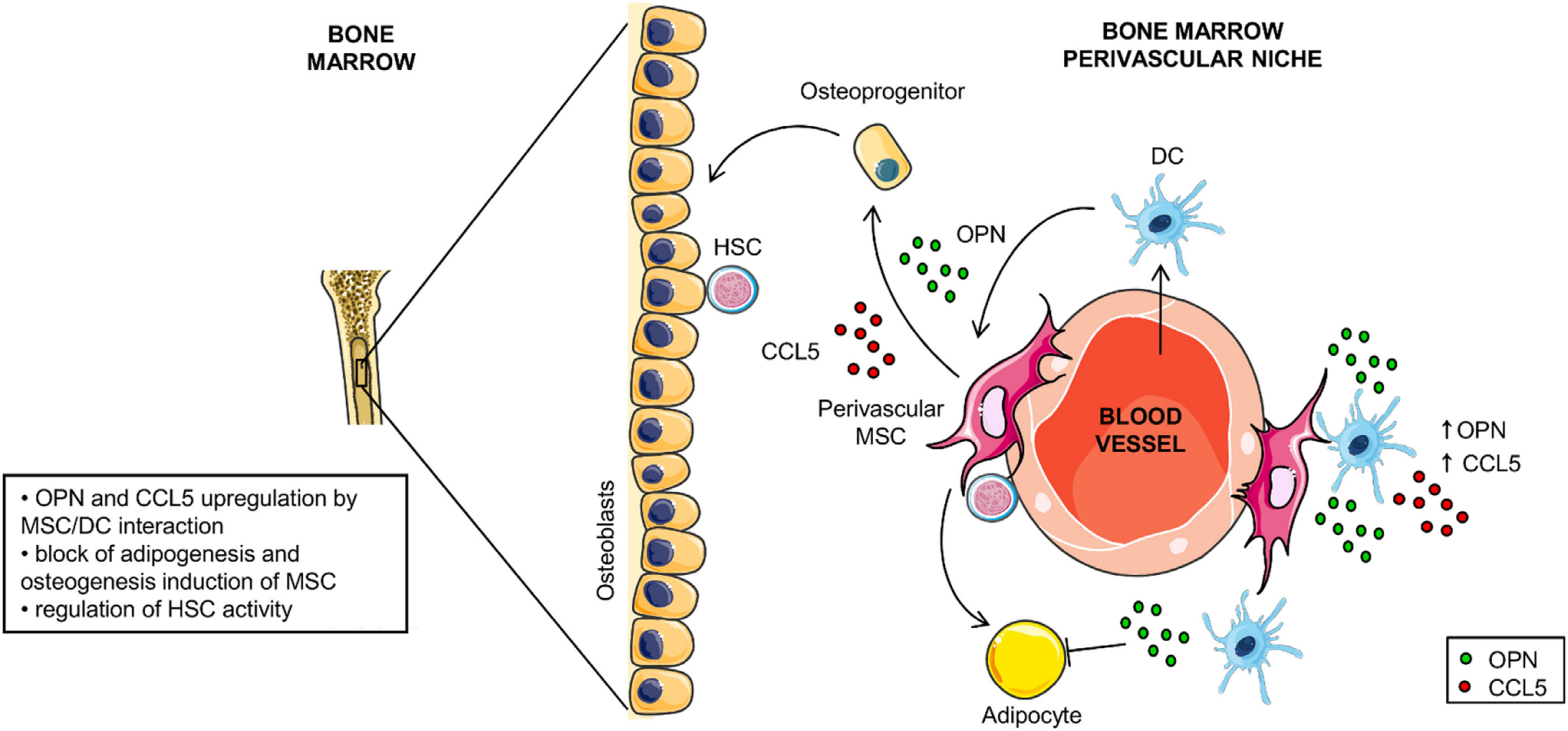
Hypothetical model for osteopontin (OPN) production in dendritic cell (DC)/mesenchymal stromal cells (MSC) cross-talk. In the perivascular area of the hematopoietic niche, DC/MSC interaction results in upregulation of OPN and CCL5 that influences the balance between osteogenesis and adipogenesis of MSCs.

Based on our data and on the data available in literature we propose two scenarios in which the opposite OPN regulation observed during the DC/MSC interplay might be relevant. The first one is at sites of inflammation where activated MSCs inhibit OPN production through the release of PGE_2_ and reduce the DC-driven proinflammatory processes. The second is the BM environment that contains resident MSCs in addition to various immune cell types, including DCs albeit in a low number (65, 66). Here, MSCs contribute to the homeostasis of the hematopoietic compartment and differentiate into osteoblasts, adipocytes and reticular cells according to local soluble factors [for review, see Ref. (31, 67, 68)]. Interestingly, OPN is a HSC niche component known to regulate the size of the hematopoietic stem cell pool (32, 67, 69, 70). We speculate that within this environment, the interplay between DCs and MSCs may contribute to the upregulation of OPN production with the consequent inhibition of MSC-derived adipogenesis and the induction of osteogenic differentiation (hypothetical model in Figure 7). Taking together, these results indicate that DC-derived OPN is finely regulated by MSCs and skew MSC differentiation.

## Ethics Statement

This study was carried out in accordance with the recommendations of “Comitato Etico Interaziendale A.O.U. Città della Salute e della Scienza di Torino—A.O. Ordine Mauriziano—ASL TO1, number 0009806” with written informed consent from all subjects. All subjects gave written informed consent in accordance with the Declaration of Helsinki. The protocol was approved by the “Comitato Etico Interaziendale A.O.U. Città della Salute e della Scienza di Torino—A.O. Ordine Mauriziano—ASL TO1.”

## Author Contributions

SaS, SiS, and TM participated in the design of the study. SaS, VS, LL, SL, GP, DA, ED, and SC participated in data acquisition and analysis. TM and SiS wrote the manuscript. GD and CC participated in data interpretation and manuscript revision.

## Conflict of Interest Statement

The authors declare that the research was conducted in the absence of any commercial or financial relationships that could be construed as a potential conflict of interest.
